# Mesodermal origin of median fin mesenchyme and tail muscle in amphibian larvae

**DOI:** 10.1038/srep11428

**Published:** 2015-06-18

**Authors:** Yuka Taniguchi, Thomas Kurth, Daniel Meulemans Medeiros, Akira Tazaki, Robert Ramm, Hans-Henning Epperlein

**Affiliations:** 1 Department of Anatomy, Technische Universität Dresden, Fetscherstrasse 74, D-01307 Dresden, Germany; 2 Center for Regenerative Therapies, Technische Universität Dresden, Fetscherstrasse 105, D-01307 Dresden, Germany; 3 Department of Ecology and Evolutionary Biology (EBIO), University of Colorado, Ramaley N122 Campus Box 334, Boulder, CO 80309-0334, USA; 4 Leibniz Research Labs for Biotechnology Artificial Organs (LEBAO),Dept of Cardiac, Thoracic,Transplantation and Vascular Surgery, MHHCarl Neuberg Str. 1, D-30625 Hannover, Germany

## Abstract

Mesenchyme is an embryonic precursor tissue that generates a range of structures in vertebrates including cartilage, bone, muscle, kidney, and the erythropoietic system. Mesenchyme originates from both mesoderm and the neural crest, an ectodermal cell population, via an epithelial to mesenchymal transition (EMT). Because ectodermal and mesodermal mesenchyme can form in close proximity and give rise to similar derivatives, the embryonic origin of many mesenchyme-derived tissues is still unclear. Recent work using genetic lineage tracing methods have upended classical ideas about the contributions of mesodermal mesenchyme and neural crest to particular structures. Using similar strategies in the Mexican axolotl (*Ambystoma mexicanum*), and the South African clawed toad *(Xenopus laevis*), we traced the origins of fin mesenchyme and tail muscle in amphibians. Here we present evidence that fin mesenchyme and striated tail muscle in both animals are derived solely from mesoderm and not from neural crest. In the context of recent work in zebrafish, our experiments suggest that trunk neural crest cells in the last common ancestor of tetrapods and ray-finned fish lacked the ability to form ectomesenchyme and its derivatives.

According to the germ layer theory of development^1^ the endoderm gives rise to the digestive tract, the ectoderm generates the nervous system and skin, while muscles and bones are derived from mesoderm. However, classical and modern studies using vital dyes, tissue grafts, and ablations showed that these boundaries are not inviolable, and an ectodermal cell population, the neural crest, generates cartilage, bone, and connective tissue in the head^2^,^3^,^4^,^5^,^6^,^7^,^8^,^9^,^10^ and median fins^4^,^5^,^6^,^11^,^12^,^13^,^14^,^15^.

While it was initially assumed that the mesenchyme populating the median fins was entirely neural crest-derived, tissue ablation and labelling in the anuran, *Xenopus laevis*^16^,^17^, indicated a mesodermal contribution. A similar result was obtained in urodeles, when Sobkow *et al.*^18^ showed that both neural folds and somites (presumably dermatome) appear to generate dorsal fin mesenchyme in axolotl larvae. More recently, Lee *et al.*^19^, used genetic lineage tracing methods to demonstrate that the paraxial mesoderm is the sole source of fin mesenchyme in zebrafish (*Brachydanio rerio*).

Like fin mesenchyme, lineage tracing experiments suggested an unexpected ectodermal source for striated muscle in the tail. Bijtel^20^ proposed that the posterior 1/5 of the neural plate (neuroectoderm) in neurulae (stage 16) generates the future tail muscles of urodele amphibian larvae. The apparently ectodermal origin of the larval tail muscles in *Xenopus* was corroborated by Tucker and Slack^21^ who grafted the “tail forming region”, the whole posterior neural plate/fold area, from FDA labeled donors (stage 18/19) into unlabeled hosts. While consistent with previous work, these experiments did not address whether the neural plate, neural crest, or some unidentified cell population in the grafts, were giving rise to this tissue.

Here we show that in axolotl and *Xenopus laevis* larvae, the median fin mesenchyme and tail muscle are of mesodermal origin. In the context of recent work in zebrafish, our results suggest that trunk neural crest cells in the last common ancestor of tetrapods and ray-finned fish lacked the ability to form ectomesenchyme and its derivatives.

## Results

### The posterior neural plate and neural folds form median tail fin mesenchyme and tail muscles

**Figure 1:**
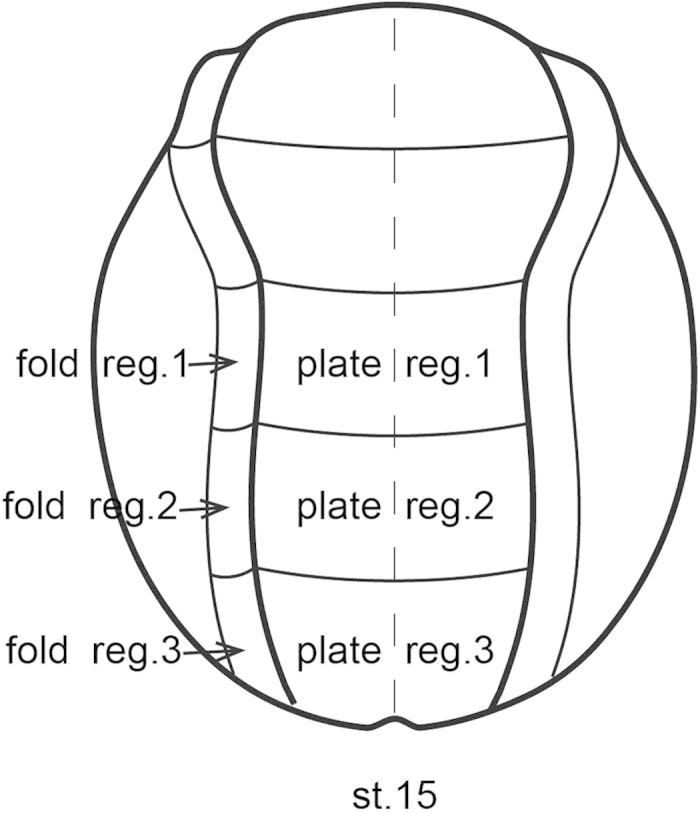
Topography of tissues used for fate mapping experiments in the axolotl neurula (stage 15). **A**, three defined neural plate areas (plate regions1–3) and neural fold areas (fold regions1–3) were grafted from GFP+ donors (stage 15) homo- or heterotopically into white (d/d) hosts (stage 15) for studying their potency to develop into striated tail muscle or fin mesenchyme. Experimental results were analyzed in larvae at stage 41 (1 cm length; details Figs. 2 and 3). Mapping was according to Bijtel^20^ who originally divided the neural plate of stage 16 neurulae along the cranio-caudal axis into 5 rectangular zones. We used stage 15 neurulae with a wider plate for better grafting. Here the length to width of each neural plate belt measures about 300 × 1000 (lxw) μm; anterior belts are wider than posterior ones. Neural fold areas on either side of the plate measure about 300 × 200 (lxw) μm. As only prospective trunk but no cranial plate was needed for grafting, we nominated the anterior trunk rectangle “region 1” (plate region1), the middle one “region 2” (plate region2) and the posterior one “region 3” (plate region3). Trunk neural fold zones are called accordingly: “left or right neural fold region 1, 2, and 3” (fold region1, -2 and -3).

**Figure 2:**
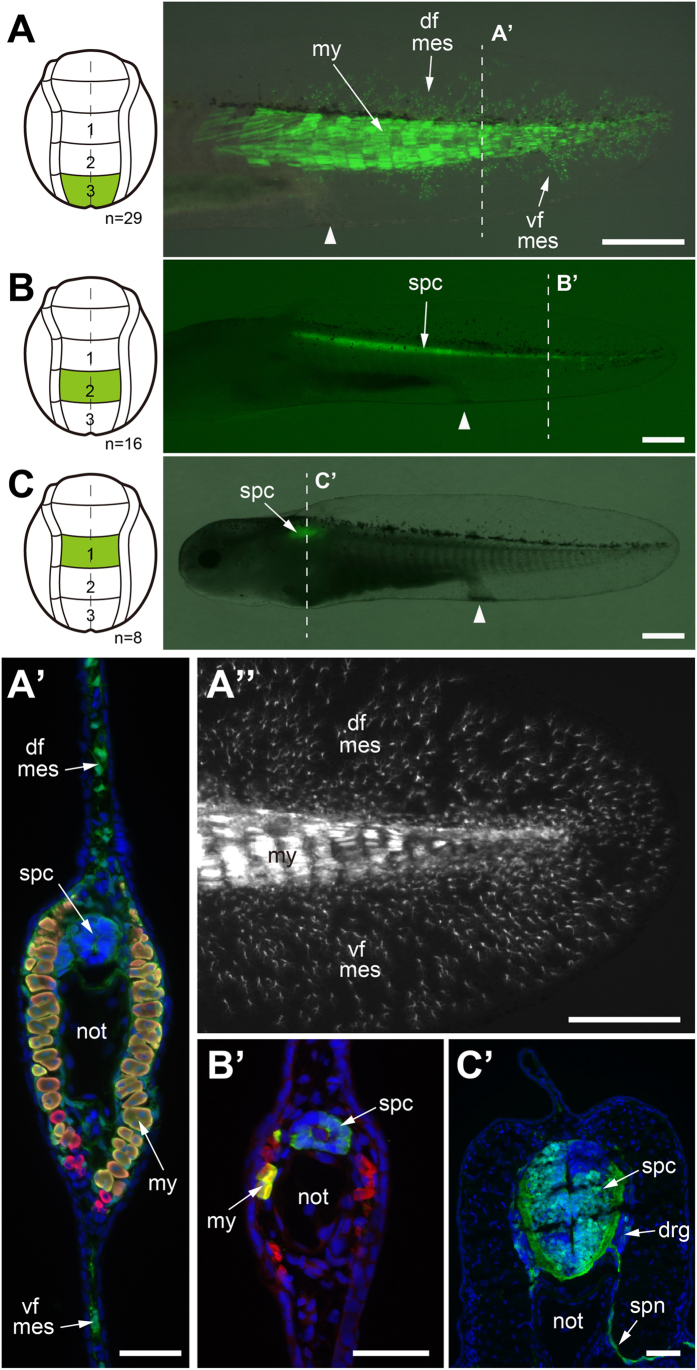
Results of fate mapping experiments of neural plate in the axolotl neurula (stage 15). Only posterior trunk neural plate regions contribute to posterior trunk/tail muscle and fin mesenchyme. **A**–**C**, homotopic transplantation of three defined GFP+ neural plate regions (accentuated in green) from a GFP+ donor (stage 15) to a white (d/d) host (stage 15) and visualization of graft-derived GFP+ cells in larvae at stage 41 (1 cm length). White arrowhead indicates position of cloaca in larvae. **A’**–**C’**, transverse cryosections through larvae shown in A–C containing GFP+ grafts; dashed lines in A–C indicate sectioning planes; sections are overlays of fluorescence images. Dapi, blue; anti-12/101 (muscle), red; anti-GFP, green. **A** and **A’**, GFP+ region3 plate gives rise to most myotome cells in the tail and posterior trunk and to mesenchymal cells of the dorsal and ventral tailfin (faintly visble). **B and B’**, GFP+ region2 plate gives rise to some cells in the spinal cord and tail myotomes. **C and C’**, GFP+ region1 plate contributes to cells in the spinal cord of the anterior trunk. **A”**, enlargement of tailfin for visualizing mesenchymal cells (mes) in the dorsal (df) and ventral tailfin (vf); animal different from that in A. Number of experiments: A, 29; B, 16; C, 8. Abbreviations: df, dorsal fin; vf; ventral fin; mes; mesenchymal cell; my, myotome; spc, spinal cord; not, notochord; drg, dorsal root ganglia; spn, spinal nerve. Scale bars, 1 mm (A–C), 100 μm (A’–C’) and 500 μm (A”).

**Figure 3:**
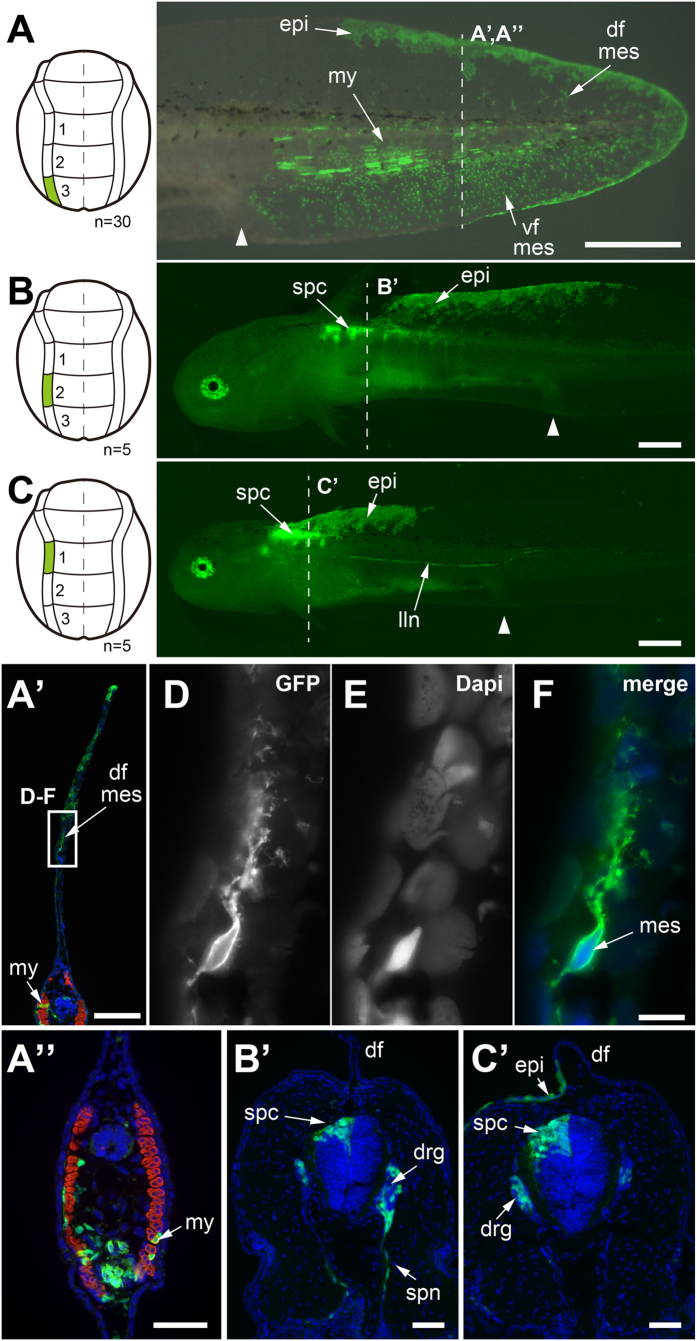
Results of fate mapping experiments of neural fold in the axolotl neurula (stage 15). Only posterior trunk neural fold regions contribute to posterior trunk/tail muscle and fin mesenchyme. **A-C**, homotopic transplantation of three defined GFP+ neural fold regions (accentuated in green) from a GFP+ donor (stage 15) to a white (d/d) host (stage 15) and visualization of graft-derived GFP+ cells in larvae at stage 41 (1 cm length). White arrowheads indicates position of cloaca in larvae. **A’**–**C’ and A”**, transverse cryosections through larvae shown in A–C containing GFP+ grafts; dashed lines in A–C indicate sectioning planes; sections are overlays of fluorescence images. Dapi, blue; anti-12/101 (muscle), red; anti-GFP, green. **A, A’ and A”**, GFP+ region3 fold gives rise to few muscle cells in tail myotomes, to few mesenchymal cells in the dorsal and ventral tailfin and to some tail epidermis (upper and lower seam of tailfin). **B and B’**, GFP+ region2 fold contributes cells to the spinal cord, dorsal root ganglia and fin epidermis in the mid trunk. The labelling of the epidermis is not visible here in B‘ but optimal further posteriorly to the ganglia (see Fig. S1). **C and C’**, GFP+ region1 gives rise to cells in the spinal cord, dorsal root ganglia, fin epidermis and to the middle lateral line nerve in the anterior trunk. **D**–**F**, higher enlargements of boxed area in A’. Presence of GFP+ mesenchymal cells in the dorsal tailfin after grafting GFP+ fold region3. Number of experiments: A, 30; B, 5; C, 5. Abbreviations: df, dorsal tail fin; vf: ventral fin; mes: mesenchymal cell; my, myotome; epi, epidermis; spc, spinal cord; lln, lateral line nerve; drg, dorsal root ganglia; spn, spinal nerve; not, notochord. Scale bars, 1 mm (A–C), 200 μm (A’), 20 μm (F) and 100 μm (A”, B’, C’).

We decided to re-examine the germ layer origins of the median fin mesenchyme and tail muscle in axolotl and *X. laevis* using genetically-labeled tissue grafts, vital dyes, and gene expression. We first grafted three defined neural plate areas (plate regions1–3) and neural fold areas (fold regions1–3) one by one from GFP+ donors (stage 15) homo- or heterotopically into white (d/d) hosts (stage 15) for studying their potency for developing into striated tail muscle or fin mesenchyme (schematics Fig. 1). These domains corresponded largely to the grafts done by Bijtel^20^ and Tucker and Slack^21^, except that we performed separate grafts for the neural plate and neural fold regions. These experiments confirmed that the neural plate and neural folds in the most posterior domain, region 3, are the exclusive source of tail fin mesenchyme and generate most of the tail muscle (Fig. 2A and A’; Fig. 3A,A’). This region also contributed some spinal cord cells (Fig. 2 A and A’) contradicting the results of Bijtel^20^. We also found that neural plate in region 2 gives rise to spinal cord cells as well as to some tail muscle cells (Fig. 2B,B’) and the neural plate in region 1 gives rise to spinal cord in the anterior trunk, but to no tail structures (Fig. 2C,C’). The neural folds of regions 2 and 1 do not contribute to tail structures or fin mesenchyme. Rather, neural fold region 2 mainly gives rise to spinal cord, dorsal root ganglia (DRG), fin epidermis (Fig. 3B,B’) and neural fold region 1 contributes to spinal cord, DRG, fin epidermis and the middle lateral line nerve in the anterior trunk (Fig. 3C,C’). Both regions 1 and 2 contribute also to pigment cells (region 1: not shown; region 2: see Fig. S1). Whereas DRG and fin epidermis derived from reg. 1 fold can be demonstrated convincingly on the transverse section through the anterior trunk (Fig. 3C), labeled fin epidermis derived from reg. 2 fold is not present on the transverse section through the labeled DRG (Fig. 3B’) and occurs only more posteriorly (Fig. 3B). Evidence for labeled epidermis in this position is provided in Fig. S1. As expected, we also found that the neural folds in region 1-2 (between zones 1 and 2) generate pigment cells (Fig. S2).

**Figure 4:**
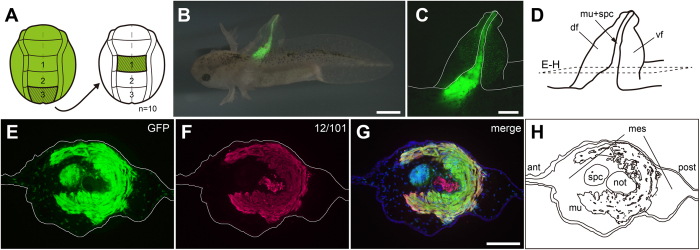
Heterotopic grafting of plate region3 into plate region 1 area gives rise to an ectopic tailfin. **A**, operation schematic. **B** and **C**, an ectopic tail is formed from the GFP+ graft in the anterior trunk (**C**, enlarged); overlay of fluorescence and bright field images. **D**, schematic indicating transverse (E–H) sectioning planes through ectopic tail. **E**, distribution of GFP+ cells derived from grafted GFP+ plate region3. **F,** myotome cells detected with 12/101 antibody. Red dot in notochord is due to unspecific staining. **G**, merged images; the ectopic tail contains GFP+ cells in spinal cord, muscle and mesenchyme but not in notochord. Number of experiments: 10. Abbreviations: ant, anterior; post, posterior; spc, spinal cord; not, notchord; mu, muscle; df, dorsal fin; vf, ventral fin; ant, anterior; post, posterior. Scale bars, 1 mm (B), 500 mm (C and G).

**Figure 5:**
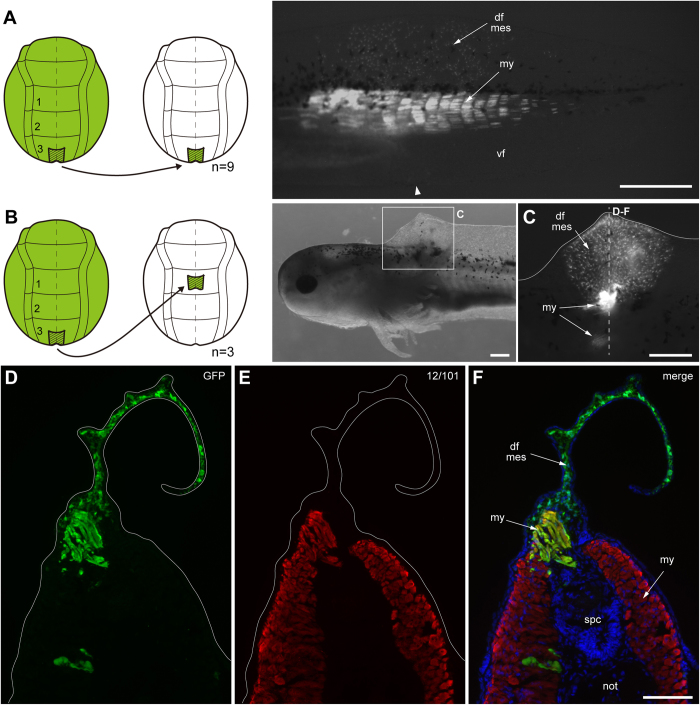
Central part of the posterior region 3 plate gives rise to fin mesenchyme and muscle. Homotopic transplantation (A) and heterotopic transplantation (B) of medial GFP+ neural plate tissue from the posterior region 3 of a GFP+ donor (stage 15) to a white (d/d) host (stage 15). **A**, visualization of graft-derived GFP+ cells in the myotomes (my) of the posterior trunk and anterior tail of a host at stage 41; dorsal fin mesenchyme (mes) is also GFP+; white arrowhead points to position of cloaca. **B**–**F**, visualization of GFP+ cells in the dorsal fin of the anterior trunk of a host at stage 41. D–F, demonstration of GFP+ fin mesenchyme (mes) and striated paraxial muscle on transverse sections (plane indicated in C). White lines in C–E help to see the outlines of dorsal parts of larvae. Number of experiments: A, 9; B, 3. Abbreviations: df, dorsal fin; vf, ventral fin; mes, mesenchyme; my, myotome; spc, spinal cord; not, notochord. Scale bars: 1 mm (A), 500 mm (B and C) and 200 mm (F).

**Figure 6:**
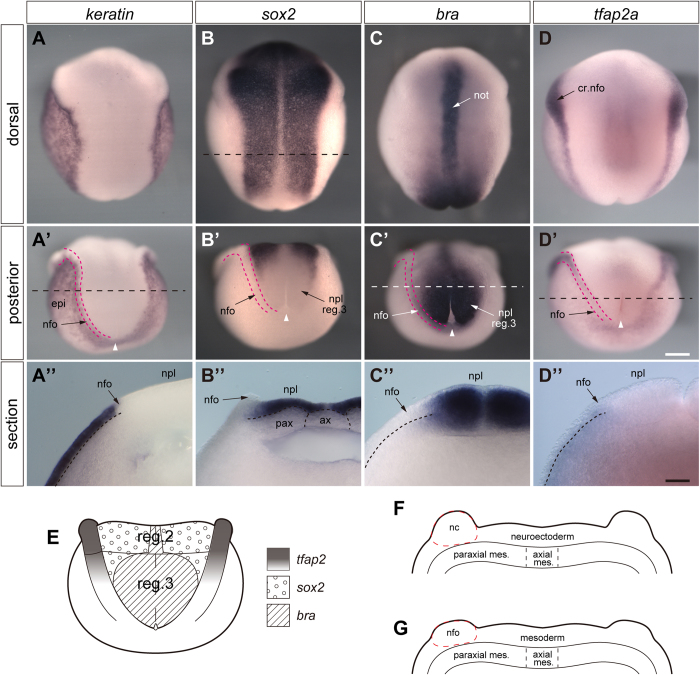
Expression of molecular markers for epidermis, mesoderm, and neural crest. *In situ* hybridization of axolotl neurulae (stage 15) with keratin (A, A’), *sox2* (B, B’ and B”), brachyury (C, C’ and C”) and tfap2a (D, D’ and D”) riboprobes. **A**–**D**, dorsal views of whole embryos. **A’**–**D’**, posterior views of whole embryos. **A”**–**D”**, posterior aspects of anterior halves of bisected embryos. Sectioning planes are indicated by dashed lines and run through the middle of region 3 fold/plate (**A’, C’** and **D’)** or through region 2 (**B**). Red double-dashed lines indicate neural folds; prospective epidermis is lateral and neural plate medial to the folds. **E**, fate of plate/fold region3 based on *in situ* hybridization with *brachyury* (*bra*), *sox2* and *tfap2a* riboprobes (neurula stage 15). *Brachyury*: positive in the centre of plate region 3; *tfap2a*: positive.in cranial and trunk neural folds until the anterior part of fold region 3; *sox2*: positive in cranial and region 2 plate. **F** and **G**, transverse sections through neural fold/plate (stage 15) in region 1–2 (F) and middle of region3 (G). Axial differences of neural plate and neural crest potential become evident (neuroectoderm vs. mesoderm and neural crest vs. neural fold, respectively). These data and the indication of the distribution of the *tfap2*-, *sox2*- and *bra*-zones in E are based on *in situ* hybridization (see above). nc in F, prospective neural crest; nfo in G, *tfap2a*-negative neural fold tissue, probably mesoderm. Number of experiments: about 20 for each riboprobe. White arrowheads in A‘-D‘ point to blastopore. Abbreviations: not, notochord; nfo, neural fold; cr. nfo, cranial neurl fold; npl, neural plate; ax, axial mesoderm; pax, paraxial mesoderm. Scale bars, 500 μm (D’) and 200 μm (D”).

Since we showed that both neural plate and neural fold in region 3 form tail muscle and fin mesenchyme, we decided to compare the relative contributions of the two regions to these derivatives. First we tested the effect of ablating region 3 neural plate and region 3 neural fold on tail morphology. We found that while ablation of region 3 neural plate results in severe tail malformation (Fig. S3A), ablation of region 3 neural fold leads to only limited defects (Fig. S3B). We then tested if neural plate region 3 was capable of organizing the formation of fin mesenchyme or a full tail at a heterotopic site where axial mesoderm and neural crest are present. We grafted region 3 plate into the position of region 1 plate and found that an almost complete ectopic tail, with fin mesenchyme, muscle, and spinal cord derived from the GFP+ graft was formed, and with notochord derived from the host (Fig. 4), as reported previously for *Xenopus*^22^. Both the mesodermal homo- and heterotopic plate region 3 grafts might contain marginal ectodermal and NC cells. To rule out any contribution of NC, we isolated only the central part of the posterior region 3 plate and grafted it homo- and heterotopically to white hosts. We observed GFP+ striated muscle and fin mesenchyme in both cases (Fig. 5). Due to its topography and as evidenced by *in situ* hybridization this central region consists definitely only of mesoderm (Fig. 6). Taken together, these experiments suggest that the posterior neural plate and neural folds (region 3) are the main source of tail fin mesenchyme and tail muscle.

### The posterior ‘neural’ plate and ‘neural’ folds are mostly mesodermal, with only a small neural crest component in the folds

Moreover, we show that within this domain the neural plate, not the neural folds, is the major source of these derivatives. While consistent with some previous work^20^,^21^, the apparent ability of both the neural plate and neural fold grafts to generate striated muscle was unusual. Therefore, we examined the developmental potential of the tissues in region 3 in detail using definitive molecular markers for mesoderm (*brachyury*), neural plate (*sox2*), epidermis (*keratin*) and neural crest (*tfap2a*) (Fig. 6). As expected, we found keratin transcripts in the epidermal component of the neural folds (Fig. 6A,A’). However, we also found that in the posterior portion of plate region 3, *sox2* was restricted to the lateral part of the neural plate, adjacent to the neural fold (Fig. 6B,B’). We also noted unexpected expression of *brachyury* throughout the centre of the posterior ‘neural’ plate and in the underlying mesoderm, including its lateral edges (Fig. 6C,C’), which are contained in the neural fold grafts. This expression appeared contiguous with *brachyury* expression in axial mesoderm, as *brachyury* mRNA was detected in cells of the chordoneural hinge. Finally, *tfap2a* labels cranial neural folds strongly, anterior and mid trunk neural folds moderately and is absent from mid to posterior trunk fold region3., suggesting little, if any, neural crest arises from this domain (Fig. 6D,D’). A synopsis of the distribution of three of the four molecular markers investigated (*brachyury*, *sox2*, and *tfap2a*) on the surface of the posterior neurula is shown in Fig. 6E while transverse sections through the anterior (Fig. 6F) and posterior prospective trunk (Fig. G) of the neurula reveal axial differences of the neural plate and neural crest potential. Our data suggest the ‘neural’ plate of region 3 is mainly mesodermal while the ‘neural’ fold grafts likely include mesoderm (*brachyury*+), neural tissue (*sox2*+), and possibly a few neural crest cells (*tfap2a*+). While *tfap2a* is a robust marker for neural crest cells, it is also expressed at low levels in the epidermal ectoderm of early neurulae. To confirm the presence of neural crest cells in region 3 and their potential participation in mesenchyme/striated muscle formation of the tail we looked for GFP+ pigment cells in white hosts receiving region 3 neural fold grafts (Fig. S4). We detected xanthophores and some melanophores when the entire region 3 neural fold was grafted (Fig. S4), but no GFP+ pigment cells when only its posterior portion was grafted (data not shown) which is *tfap2a*-negative and probably mesodermal tissue. As all pigment cells in the vertebrate trunk are neural crest-derived, this strongly suggests some neural crest cells are present in the anterior portion of the region 3 neural fold, but absent from the posterior portion.

### Clonal analyses suggest mesodermal origins for most, or all, fin mesenchyme and tail muscle

**Figure 7:**
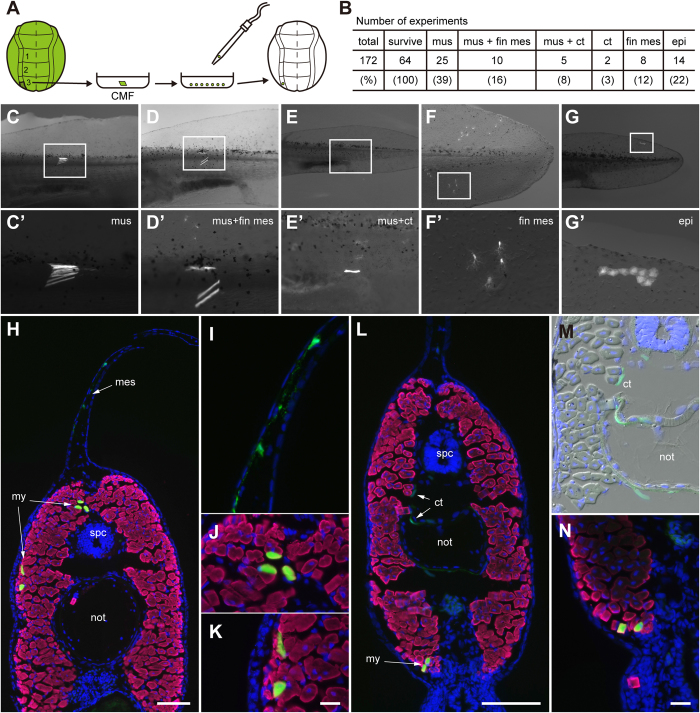
Grafted single GFP+ neural fold reg3 cells reveal mesodermal but no pigment cell traits in the host tailfin. **A**, Schematic of experimental procedure. Single neural fold cells were isolated from a dorsal neural fold explant (left middle to posterior region3) of a GFP+ donor (stage 15) and grafted homotopically into a white host (stage 15). **B**, summary of experimental results; frequency of GFP+ cells in tail tissues of host larvae (stage 41, 1 cm); tissues: mus, muscle; fin mes, fin mesenchyme; ct, connective tissue; epi, epidermis. **C**–**G**, types and frequency of GFP+ tissues that had developed from a single grafted neural fold region3 GFP+ cell in tail regions of living axolotl hosts (stage 41, 1 cm); bright field/FITC. **C’**–**G’,** higher enlargements of boxed areas in C–G. **C** and **C’**, only muscle cells (39%); **D** and **D’**: muscle and fin mesenchyme cells (16%); **E** and **E‘**: muscle and connective tissue (8%); as the connective tissue is located inside the larva it is dim and insharp in wholemount images; **F** and **F’**: only fin mesenchyme (12%); **G** and **G’**: epidermis (22%). **H** and **L**, Transverse sections through tails of larvae shown in D and E, respectively. **I**–**K**, higher enlargement of GFP+ fin mesenchymal and muscle cells from H. **M**–**N**, higher enlargement of connective tissue (M) and muscle cells (N) from L . **H**–**L** and **N**, merged images of GFP, 12/101 (red) and Dapi. **M**, merged image of GFP, bright field and dapi. Scale bars, 200 μm (H and L) and 50 μm (K and N). I–K and M–N have the same magnification.

We next sought to determine the proportion of fin mesenchyme and tail muscle derived from mesoderm, and the fraction derived from neural crest. We removed the dorsal fold tissue to be disaggregated from a middle to posterior location of fold region 3 (Fig.7A). We assume that this area consists of prospective epidermis, neural crest and of mesoderm (from the lateral neural plate). Based on previous results of grafting the entire region 3 fold we could expect that single cells grafted from region 3 fold will give rise to muscle, mesenchyme (see Fig. 3A) and pigment cells (Fig. S4). The single cells produced from dorsal neural fold cell aggregates were transferred into stage-matched white hosts and allowed to differentiate. We reasoned that if GFP+ fin mesenchyme or tail muscle cells were observed in the same larvae as GFP+ pigment cells, they must be neural crest-derived. Alternatively, if GFP was only seen in mesodermal derivatives like striated muscle, fin mesenchyme, and connective tissue surrounding the myotomes, the progeny of transplanted cells were likely to be mesoderm-derived. These experiments are summarized in Fig. 7. Out of 64 individually grafted cells, we did not observe any GFP+ pigment cells, neither alone nor together with fin mesenchyme or muscle in the same host. While this does not rule out the possibility that neural crest cells make a nominal contribution to fin mesenchyme and/or tail muscle, it strongly suggests that most, or all, these tissues are mesoderm-derived. One main reason for the absence of pigment cells from singly grafted neural fold cells could be that no or only few NC cells occur in fold region 3. This is supported by the observation that when the posterior part of fold reg. 3 is grafted no GFP+ pigment cells develop in the host (5 experiments; data not shown). Further support comes from the *in situ* hybridization experiments with tfap2 which is absent from mid to posterior fold region3 (Fig. 6D’). Thus, pigment cells would not be expected to develop from single cells of that area. Another possible reason for their absence is that some prospective pigment cells died during the grafting or later in the host. In contrast, when the entire reg. 3 is grafted GFP+ pigment cells develop in the host (Fig. S4). The latter case can be explained by assuming that pigment precursor cells were present only in the anterior part of fold region 3 or at the border to fold region 2 where tfap2 is slightly positive. In summary, single cell grafting has shown that only mono- or bipotent derivatives developed from mid to posterior areas of fold region 3 which are very likely of mesodermal origin. Because no pigment cells were observed a neural crest contribution to fin mesenchyme can nearly be ruled out.

### Lineage tracing in Xenopus larvae reveals mesodermal origin of fin mesenchyme

**Figure 8:**
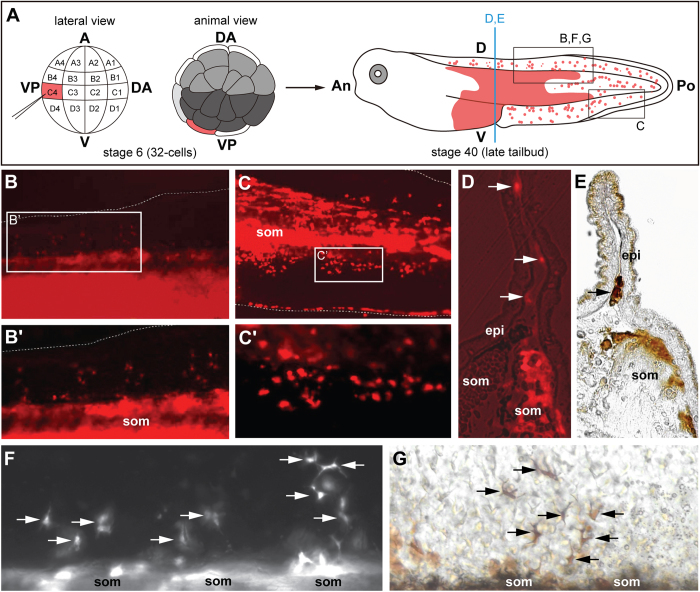
Descendants from blastomere C4 form fin mesenchyme in *Xenopus laevis*. **A**, Schematic of the experiment. At the 32-cell stage one C4 blastomere was labelled by microinjection of ruby-dextran. At stage 40 the labelling can be found in somites, in the posterior part of the intestine, in the blood and in the fin. The squares indicate the areas displayed in B,C,F,G. The vertical line indicates the cutting planes in D and E. **B**,**B’C**,**C’**, Groups of labelled cells migrate from the somites to the dorsal (B,B’) and ventral fin (C,C’). Squares in B and C indicate the regions displayed in B’ and C‘ at higher magnification. **D**, Transversal section through posterior trunk with labelled somite (som) and fin mesenchymal cells (arrows); overlay of dextran-fluorescence and phase-contrast image. **E**, Transversal vibratome section through posterior trunk with labelled somite (som) and with a labelled fin mesenchyme cell (arrow). DAB-peroxidase staining using the biotin-residues of the ruby-dextran tracer. **F**–**G**, Fin mesenchyme cells (arrows) at higher magnification; F, dextran fluorescence; G, after DAB-peroxidase staining. Abbreviations: A, animal; V, vegetal; DA, dorsoanterior; VP, ventroposterior; An, anterior; D, dorsal; Po, posterior; V, ventral; epi; epidermis; som, somite; Number of cases: 65

Our results show that fin mesenchyme and tail mesoderm are derived from a mesodermal population morphologically contiguous with the posterior neural plate with little, or no contribution from neural crest cells. We thus decided to corroborate the mesodermal origin of fin mesenchyme using lineage tracing in another amphibian, *Xenopus laevis*. *Xenopus* is particularly well suited to such experiments, because, unlike axolotl, a modern high-resolution fate map has been generated^23^,^24^. In *Xenopus*, descendants of the most ventroposterior C-tier cells (C4) of the 32-cell embryo eventually populate posterior tissues including the somites, but not neural tube and/or neural crest^23^,^24^. Ruby-dextran solution was used as a tracer (Fig. 8A). This lineage tracer is also biotinylated and allows alternative signal detection with avidin-peroxidase complexes and diaminobenzidine (DAB). We injected C4 blastomeres with 1–2 μl of ruby-dextran and monitored for proper C4-pattern at gastrula, neurula and early tailbud stages, selecting embryos with strong somite but no neural tube staining. This approach allows the selective analysis of somite-derived fin mesenchymal precursor cells. From 9 independent experiments 65 embryos survived the procedure and displayed the typical C4-pattern with labeled posterior somites as an important hallmark. At stage 40 the embryos were again analyzed by fluorescence microscopy. We clearly observed C4-descendant cells migrating from the somites to the dorsal fin (Fig. 8B,B’) and to the ventral fin (Fig. 8C,C’). To further characterize these cells, the larvae were fixed and imaged (Fig. 8D–G), using either the dextran fluorescence (Fig. 8D,F) or labeling of the biotin residues with avidin-biotin-peroxidase complexes and DAB (Fig. 8E,G). We could observe groups of C4-descendants in the dorsal and ventral fins at the level of the C4-labelled somites. These cells display a fibroblast-like morphology typical of fin mesenchymal cells. In these sectioned embryos we never observed C4 descendants in the neural tube but always in the somites and in the dermomyotome of the somites. Therefore, the paraxial mesoderm is a major source for fin mesenchyme in *Xenopus*.

### Cranial neural crest can form fin mesenchyme when grafted into the trunk

Our results in axolotl and *Xenopus* suggest that trunk neural crest does not contribute to fin mesenchyme in two major amphibian taxa. This could be due to some intrinsic property of trunk neural crest or some extrinsic property of the fin environment. We decided to test this by replacing region 3 neural fold of a white axolotl host with a cranial neural fold fragment of a GFP+ axolotl donor (Fig. S5 A–G’). Strikingly, we observed GFP+ mesenchyme in both the dorsal and ventral tailfins of donor larvae, suggesting 1) cranial neural crest has the capacity to form fin mesenchyme, and 2) the median fin environment is capable of supporting neural crest migration and differentiation into ectomesenchyme. The ability of cranial neural crest to form fin mesenchyme raises the possibility that cranial neural crest could also populate median fins, at least in the anterior trunk, by migrating posteriorly. To test this we replaced one cranial neural fold of a white host, with one cranial neural fold of a GFP expressing donor. To ensure that our graft did not contain any cranial mesoderm, we isolated the cranial neural folds in 4x strength Steinberg salt solution, which enables clean separation of cranial neural folds from underlying mesoderm. We observed no GFP+ mesenchyme in the fins of the host, whereas the gill mesenchyme was abundantly labeled (Fig. S5H–L).

## Discussion

In light of recent work in zebrafish, our results suggest that fin mesenchyme in the last common ancestor of all modern bony fish (ray-finned and lobe-finned fish) was derived entirely from posterior mesoderm. Similarly, a recent study has shown that mesoderm-derived mesenchyme, rather than neural crest cells, contribute to scales in the trunk of teleost fish^25^. Taken together, these results imply that trunk neural crest in modern bony fish completely lack ectomesenchyme potential. Whether this reflects the ancestral state for jawed vertebrates is unclear. Vital dye labeling and neural tube ablations suggest that fin mesenchyme in the jawless vertebrate, lamprey, is at least partly neural crest-derived^15^,^26^. If this is the case, trunk neural crest cells must have lost the ability to form fin mesenchyme at some point in the bony fish lineage. Why this difference in trunk and cranial neural crest potential arose, and why it has been maintained, is an open question. Our experiments showing that cranial neural crest can contribute to trunk fin mesenchyme suggests that some intrinsic developmental constraint prevents trunk neural crest from forming ectomesenchymal derivatives in bony fish.

It has been proposed that the ectomesenchymal cranial neural crest constitutes a cell population developmentally and evolutionarily distinct from non-ectomesenchymal cranial neural crest cells^27^,^28^,^29^. In this scenario, the difference in cranial and trunk neural crest developmental potential actually reflects the evolution of another cryptic neural crest cell population in the head, rather than a loss of developmental potential in the trunk. In axolotl, we have never observed any evidence for such a bipartite cranial neural crest. Indeed, in axolotl, as in other vertebrates, even very small grafts of tissue from the cranial neural folds consistently give rise to both ectomesenchymal and non-ectomesenchymal derivatives. Furthermore, abundant gene expression data fails to identify a distinct ectomesenchymal cranial neural crest population. Instead, a deeply conserved suite of genes is co-expressed in cranial neural crest cells before, during and after migration, with a majority of these genes also marking trunk neural crest. Thus, it is more likely that the evolution of trunk neural crest involved a loss of ectomesenchymal potential, perhaps by modifications to the neural crest gene regulatory network. Future studies in basal gnathostomes, like elasmobranchs, would help resolve precisely when these changes evolved.

## Methods

### Animal experiments

All animal procedures were performed according to the European Community and local ethics committee guidelines. All experimental protocols were approved by the Department of Anatomy and the Center for Regenerative Therapies, Technische Universität Dresden.

### Axolotl Embryos

Transgenic (GFP+) and white mutant (d/d) embryos of the Mexican axolotl (*Ambystoma mexicanum*) were obtained from our axolotl colony in Dresden, Germany. Transgenic embryos were used as donors for grafting cell and tissue fragments into white hosts. Both types of embryo have a white mutant background which prevents the migration of neural crest derived melanophores and xanthophores under the flank epidermis. White mutants are therefore much more suitable for tracing fluorescent cells than wild-type (dark) embryos (D/−) whose flanks become richly pigmented^30^. Before the operations embryos were kept in tap water at room temperature or at 7–8 °C. When they had reached late gastrula/early neurula stages (stages 12–13), they were thoroughly washed with tap water and dejellied in large plastic dishes containing a salt solution (1x Steinberg solution^31^) with antibiotics (Antibiotic-Antimycotic; Invitrogen, Karlsruhe, Germany; Ciprobay, Bayer, Leverkusen). After dejellying, fluorescent and non-fluorescent embryos were sorted under UV and stored at 7–8 °C. Stages suitable for operations were used the same or next day. Staging was according to Bordzilovskaya *et al.*^32^.

### *Xenopus* embryos and microinjection

Adult *Xenopus laevis* females were primed by injection of 100U pregnant mares serum gonadotropin (pmsg) 5 days prior to induction of superovulation using 500 units human chorionic gonadotropin (hcg). Eggs were fertilized *in vitro* according to Fey and Hausen^33^, kept in 0.1 MBSH (MBSH: 88 mM NaCl, 1 mM KCl, 2.4 mM NaHCO_3_, 0.82 mM MgSO_4_, 0.41 mM CaCl_2_, 0.33 mM Ca(NO_3_)_2_, 10 mM HEPES (pH 7.4), 10 μg/ml streptomycin sulphate and penicillin) and staged according to Nieuwkoop and Faber^34^. At the 4-cell stage eggs were dejellied with 2% cysteine, pH 8.0 and selected for dorsoanterior-ventroposterior pigmentation differences as described previously^23^,^35^. At the 32-cell stage regularly cleaving embryos were transferred to a new petridish containing 4% Ficoll-400 (Sigma, Munich, Germany) in 2/3 MBSH. 5 ng Rhodamin labelled dextran (with additional biotin residues) were injected into blastomere C4. After 3–4 hours of recovery in injection medium embryos were transferred into 0.1 MBSH and further cultivated at room temperature, protected from light. During development the injected embryos were scored for proper C4 pattern and those embryos were selected that display strong somite pattern. Whole larvae were analyzed using a Zeiss AxioImager A1 fluorescence microscope. After whole-mount analysis, the embryos were fixed and processed for histological analysis of vibratome or resin sections. Some samples were stained with avidin-biotin-peroxidase complexes and diaminobenzidine (DAB) (Vectastain elite ABC-Kit, Vector Laboratories, Burlingame, CA, USA).

### Transgenesis

The generation of transgenic animals that ubiquitously express GFP under the control of the CAGGS promotor has been described^18^.

### Embryonic operations

#### Terminology

For grafting neural plate and neural fold tissue fragments, we followed a schedule given by Bijtel^20^. Instead of using stage 16 we used stage 15 because at this earlier stage the neural plate zones are wider which facilitates grafting of plate and fold material. We divided the entire plate and the adjacent folds into 5 rectangular, belt-like zones (Fig. 1). The length of each belt amounts to about 1/5^th^ of the entire anterior-posterior axis of the neurula. The most posterior neural plate belt is roughly 300 × 1000 μm (length to width). Anterior belts are wider than posterior ones. The rectangular neural fold areas on either side of the plate belts measure about 300 × 200 μm (length to width). Our most posterior belt explant reached to the blastopore but, in contrast to Bijtel’s explant, did not include the ectoderm around it. Because we needed only trunk neural plate belts for grafting, we nominated the first one “region 1” (plate region1; anterior trunk), the middle one “region 2” (plate region2; mid trunk) and the posterior one “region 3” (plate region3; posterior trunk). Trunk neural fold zones are called accordingly: “left or right neural fold region 1, 2, and 3” (fold region1, -2 and -3).

#### Trunk neural plate/fold

Neural plate and fold areas as indicated in Fig. 1 and a medial part of posterior reg.3 plate (Fig. 5) were grafted from GFP+ donors (stage 15) into unlabeled hosts (d/d) of the same age in order to reveal the potential of these areas for tail muscle and fin mesenchyme formation. For the operations, a dejellied GFP+ donor and a white host neurula were transferred to a small agar dish (2% agar in tap water) filled with sterile 4x Steinberg solution. Each embryo was arrested in a small deepening of the agar surface and operations were carried out with tungsten needles. The hypertonicity of the salt solution facilitates tissue separation (particularly between neuroectoderm and mesoderm or mesoderm and endoderm). Immediately after the operation the hypertonic saline was diluted to 1x strength with distilled water. Operated neurulae were grown until stage 41–43 (1–1.2 cm) and processed further (sectioning, immunostaining etc.). Whole mount images were acquired with Olympus SZX-9 or SZX-16 microscopes. Schematics of operations and their results are contained in the respective figures. Numbers of operations are indicated at the schematics of each figure and in the figure legends.

#### Cranial neural fold

Like trunk also cranial neural fold portions were grafted from GFP+ donors to white (d/d) hosts. Operations were performed as for trunk neural fold portions in 4x Steinberg solution to obtain a perfect separation of fold tissue from underlying cranial mesoderm. Experiments with cranial folds should show whether cranial neural crest can form fin mesenchyme in a trunk location. First, a left cranial neural fold fragment containing cranial neural crest for the mandibular/hyoid arch was grafted heterotopically into a left host region 3 fold. Here we investigated whether cranial neural crest cells can give rise to fin mesenchyme. In another experiment the entire left cranial fold fragment was excised and implanted homotopically into a white host. With this experiment we wanted to find out whether at the head to trunk transition zone GFP+ neural crest cells migrate out and give rise to mesenchyme in the trunk dorsal fin of the host. Conditions for operations, further processing and imaging were the same as for trunk tissue grafts.

### Cryosectioning and immunostaining

Axolotl embryos and larvae were fixed in fresh 4% paraformaldehyde (PFA) in 0.1M phosphate buffer (ph 7.0) overnight at 4 °C, washed in PBS, incubated in 10% and 20% sucrose/1x PBS overnight at 4 °C, and then in 20% sucrose/ 3.5% gelatin (Bloom 80-120, Merck) / 1x PBS overnight at 37 °C and frozen in 20% sucrose/ 7.5% gelatin/ 1x PBS. In those larvae where the distribution of GFP+ cells that had migrated out from the tissue graft could not be discriminated from outside, cryosections (20–25 μm) were cut through the tails. Cryosections were stained with primary antibodies against GFP (A11122, Invitrogen) and 12/101 (Kintner and Brockes^36^). Anti-rabbit Alexa 488, Alexa 647 and anti-mouse Cy3, and Alexa 647 were used as secondary antibodies. All sections were stained with Dapi, mounted in glycerol-PBS (1:1) or Mowiol mounting medium.

### Visualization of xanthopores

To identify GFP+ xanthophores and distinguish them from unlabelled xanthophores and mesenchymal cells in the dorsal fin of larvae, these cells were identified by the presence of their pterins. Pterines were liberated from the pterinosomes of xanthophores in anaesthetized embryos with diluted ammonia (0.1–0.15%; about pH 11) as described by Epperlein and Claviez^37^ and visualized under UV where they show a bright blue fluorescence.

### Single cell grafting

Dorsal neural fold tissue of approximately 50 cells was excised from a middle to posterior location of the left fold region3 in GFP+ neurulae (stage 15) and disaggregated into single cells with Ca_2_+/Mg_2_+ free Niu-Twitty solution^38^ in a culture dish coated with 0.1% BSA (Fig. 7A). Dissociation into single cells was achieved with a mouth pipette (modified from Kurimoto *et al.*^39^). Dissociated single cells were kept in Niu-Twitty solution at room temperature until grafting was finished (maximally 2–3 h). For grafting, vital single cells were randomly picked up with a mouth pipette and transferred homotopically and isochronically (one each) into white hosts in order to investigate their potency (Fig. 7A).

### Whole-mount immunolabelling and resin embedding

Whole-mount immunolabelling and embedding into the methacrylate resin Technovit 7100 (Heraeus-Kulzer, Wehrheim, Germany) was performed as described^40^. In brief, embryos and larvae were fixed in 4% buffered PFA followed by postfixation in Methanol/DMSO^41^. After rehydration in a graded series of methanol and PBS, the samples were blocked in 20% normal goat serum in PBS and incubated with primary antibodies (rabbit anti-GFP: TP 401 from Torrey Pines; mouse anti-GFP: clone 3E6, Invitrogen; rabbit anti β-catenin: P14L^42^), followed by washes in PBS and incubation with Alexa488- and/or Alexa555-coupled secondary antibodies. After staining, the samples were postfixed in 4% PFA, followed by facultative embedding into 2% agarose/PBS to facilitate proper sample orientation later during resin embedding. Finally, samples were dehydrated in a graded series of ethanol and infiltrated and embedded in Technovit 7100. Consecutive sections (2 μm) were collected on separate slides for standard histology (1% toluidine blue / 0.5% Borax) and fluorescence (mounting with Mowiol/DABCO), respectively. This way, tissue organization of one section can be correlated to protein expression on the next section which is only 2 μm apart^40^. Images were taken on a Keyence Biozero 8000 fluorescence microscope.

### Whole-mount *In situ* hybridization

Whole-mount *in situ* hybridization was perfomed as described previously^43^. Albino or white embryos were fixed overnight at 4 °C in MEMFA. Hybridization was performed at 65–70 °C using following riboprobes: *keratin*, *sox2*, *brachyury* and *tfap2a*. White embryos were postfixed in Bouin’s fixative after NBT/BCIP detection, bleached as described^44^ and photographed with an Olympus SZX9 microscope.

## Additional Information

**How to cite this article**: Taniguchi, Y. *et al.* Mesodermal origin of median fin mesenchyme and tail muscle in amphibian larvae. *Sci. Rep.* **5**, 11428; doi: 10.1038/srep11428 (2015).

## Supplementary Material

Supplementary Information
