# Supplementary material for: Mesodermal origin of median fin mesenchyme and tail muscle in amphibian larvae
Source: Sci Rep. 2015 Jun 18;5:11428. doi: 10.1038/srep11428 (PMC4471895; doi:10.1038/srep11428)
Supplement: Supplementary Information [file srep11428-s1.doc]

**Supplementary Figures**

**Mesodermal origin of median fin mesenchyme and tail muscle in amphibian larvae**

Yuka Taniguchi^1,2,*^, Thomas Kurth^2^, Daniel Meulemans Medeiros^3^ , Akira Tazaki^2^, Robert Ramm^2,4^ and Hans-Henning Epperlein^1,2^


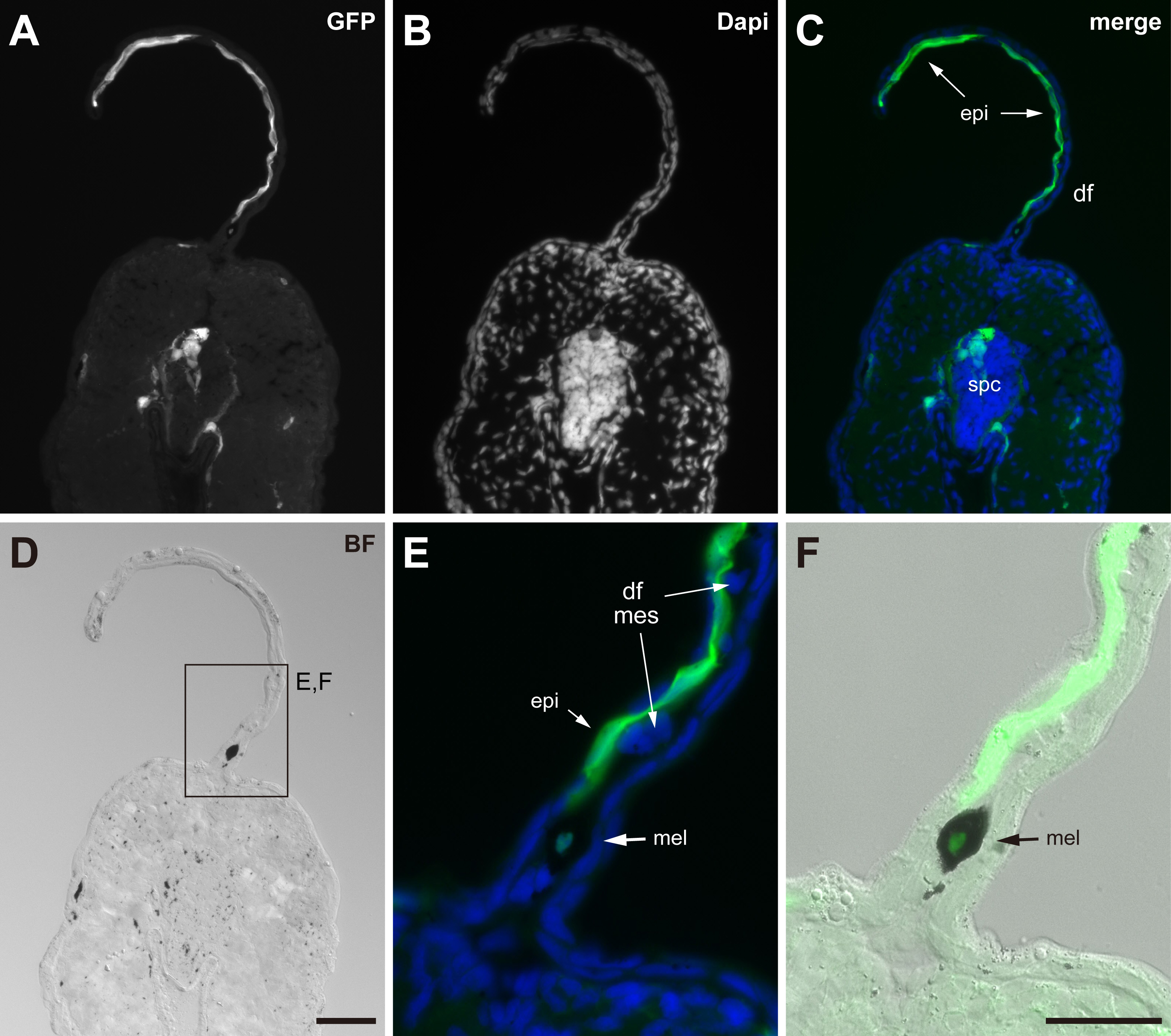


**Fig. S1:** Transverse section through the anterior trunk (region 2) providing evidence for GFP+ epidermis in the dorsal fin (df) and GFP+ pigment cells. Labelled epidermis was not visible in Fig. 3B`, because the section for Fig. 3B` was cut anteriorly to the section in the supplementary Fig. S1A-C. In this section labelling of the epidermis is optimal. **A-C**, GFP+ epidermis in the dorsal fin. **D,** bright field image of A-C. **E** and **F**, higher enlargements of boxed area in D. Mesenchymal cells in the dorsal fin (df) are GFP negative (mes, E) and pigment cells (melanophores, mel, E,F) are GFP+. Number of experiments; 5. Abbreviations: df, dorsal fin; epi, epidermis; spc, spinal cord; mes, mesenchymal cell; mel, melanophore. Scale bars: 100 µm (D) and 50 µm (F).


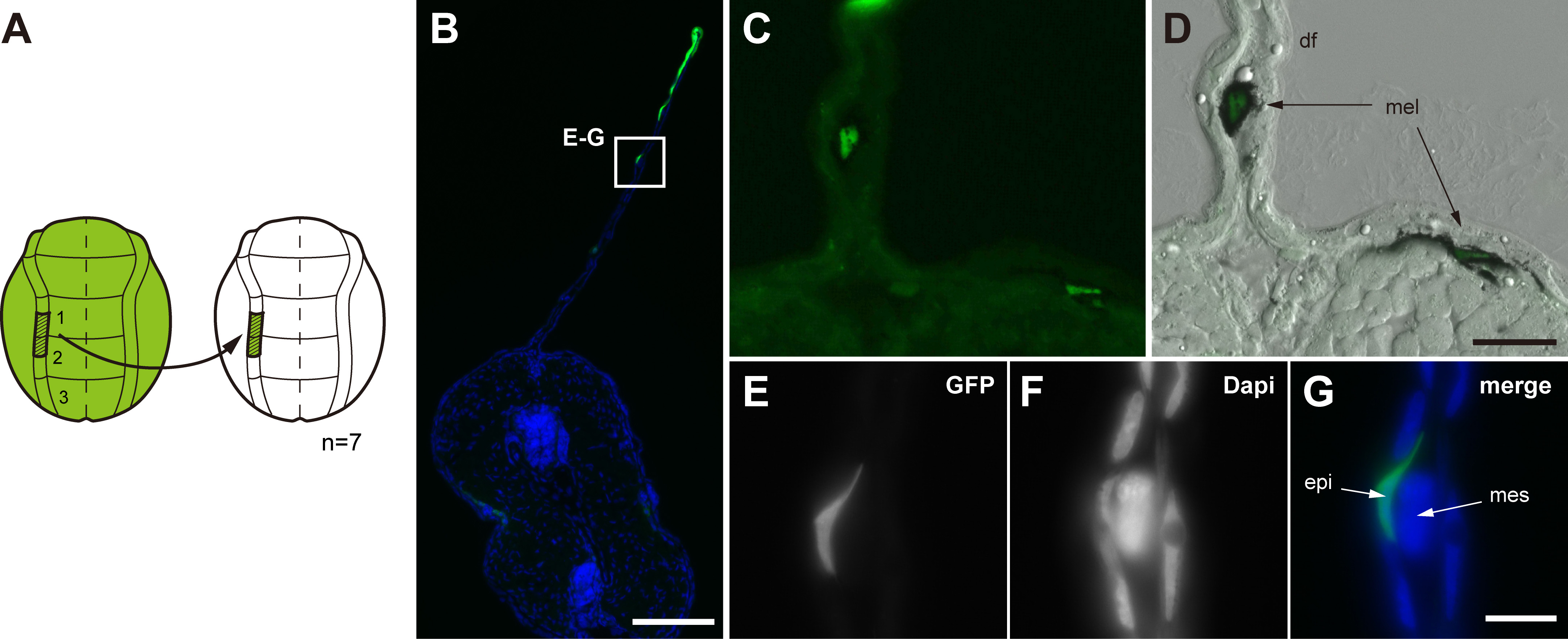


**Fig. S2: Trunk neural fold region 1-2 has a potential for pigment cells but not for fin mesenchyme.** **A**, operation schematics; homotopic grafting of fold region 1-2 (between zones 1 and 2) into white hosts (stage 15). **B**, transverse section through the mid trunk showing dorsal trunk and median fin. **C and D**, GFP+ melanophores (arrows in D) in the dorsal fin and dorsolateral mid trunk after grafting GFP+ fold region 1-2. **E**-**G,** higher enlargements of boxed areas in B. Fin mesenchyme is GFP negative. Number of experiments: 7. Abbreviations: df, dorsal fin; epi, epidermis; mes, mesenchymal cell. Scale bars, 200 µm (B), 50 µm (D) and 20 µm (G).


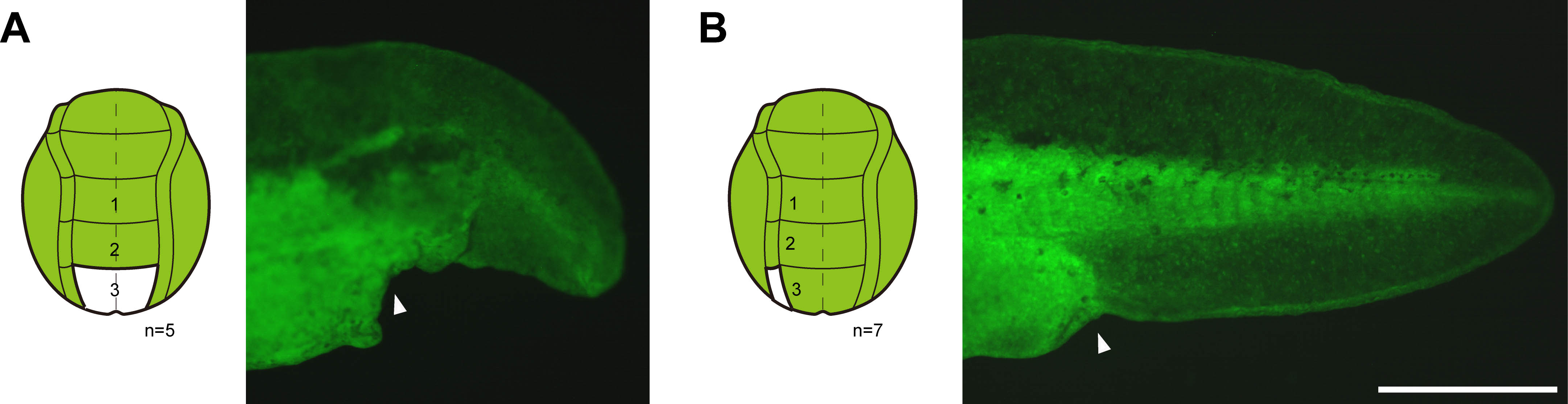


**Fig. S3: Ablation of region 3 neural plate and fold.** GFP+ donor embryos for plate region 3 (A) and fold region3 grafts (B) were analyzed in a larva at stage 38. **A,** Ablation of plate region 3 results in severe tail malformation. **B**, Ablation of fold region3 shows only minor defects (slightly shorter tail). White arrowhead points to position of cloaca. Number of experiments: A, 5; B, 7; Scale bar, 1 mm


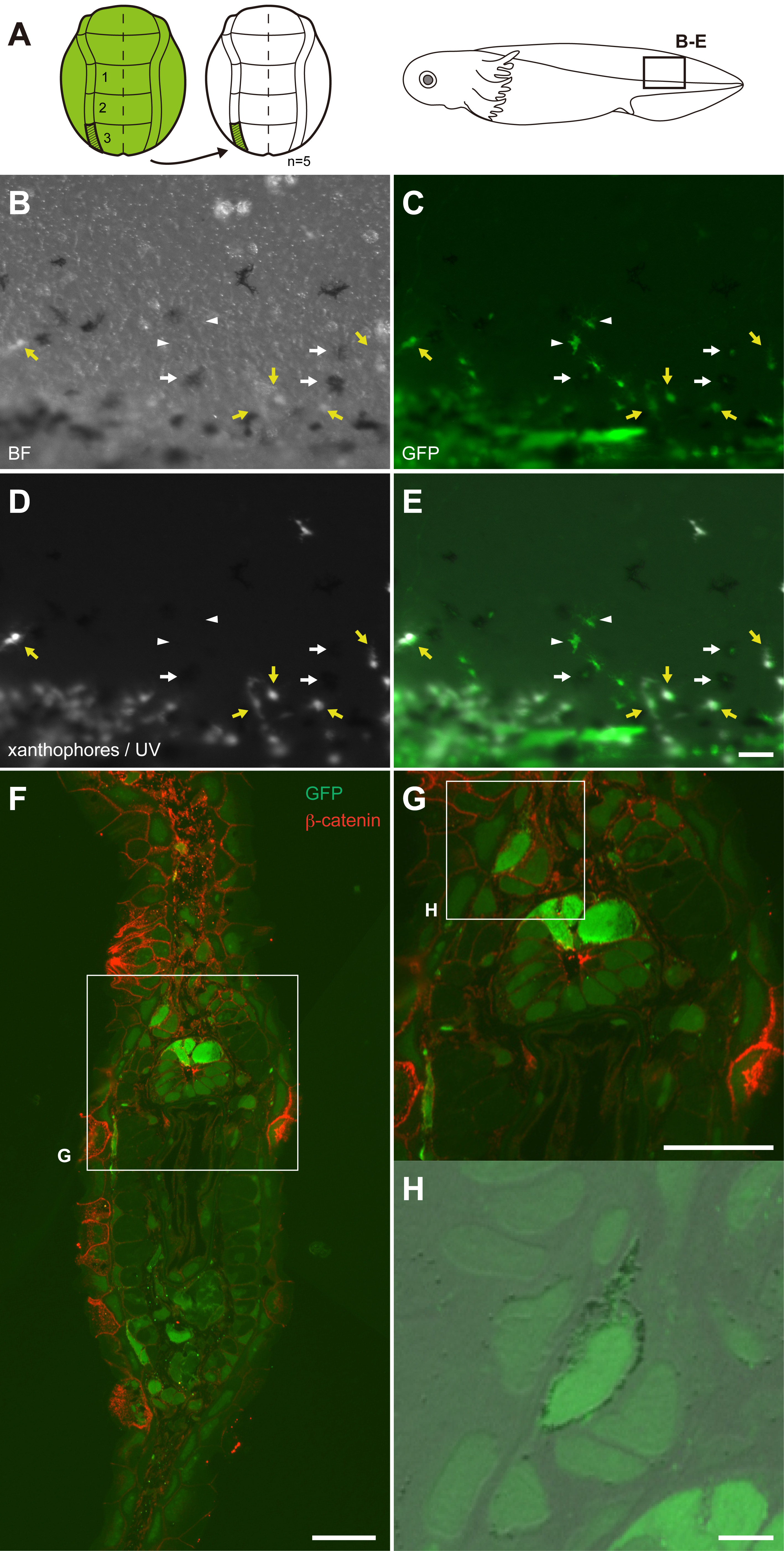


**Fig. S4: Trunk neural fold region3 has a potential for pigment and mesenchymal derivatives in the tail. A**, a left GFP+ fold region3 was grafted homotopically into a white host; the presence of pigment cells (melanophores, xanthophores) and mesenchymal cells was analyzed in larvae (stage 41). **B-E**, High resolution LM images of living dorsal tailfin. **B**, brightfield; melanophores are clearly visible as black stellate cells; 3 white arrows point to 3 melanophores that are GFP+ as revealed under UV/FITC filter in **C** (hardly visible). Xanthophores are visible with difficulty as bright cells; 5 yellow arrows point to GFP+ labeled xanthophores that are identified as blue fluorescing cells under UV with ammonia in **D** and as GFP+ labeled cells with UV/FITC in **C**; mesenchymal cells are invisible; 2 white arrowheads point to GFP+ mesenchymal cells that are visible with UV/FITC in C; **E**, overlay of B and C. **F,** transverse section through tail of host larva (stage 41) containing a GFP+ neural fold region3 implant and implant - derived GFP+ melanophores. **G** and **H**, higher enlargements of an implant-derived GFP+ melanophore. Number of experiments: 5; Scale bars, 100 µm (E), 50 µm (F, G) and 10 µm (H).

**
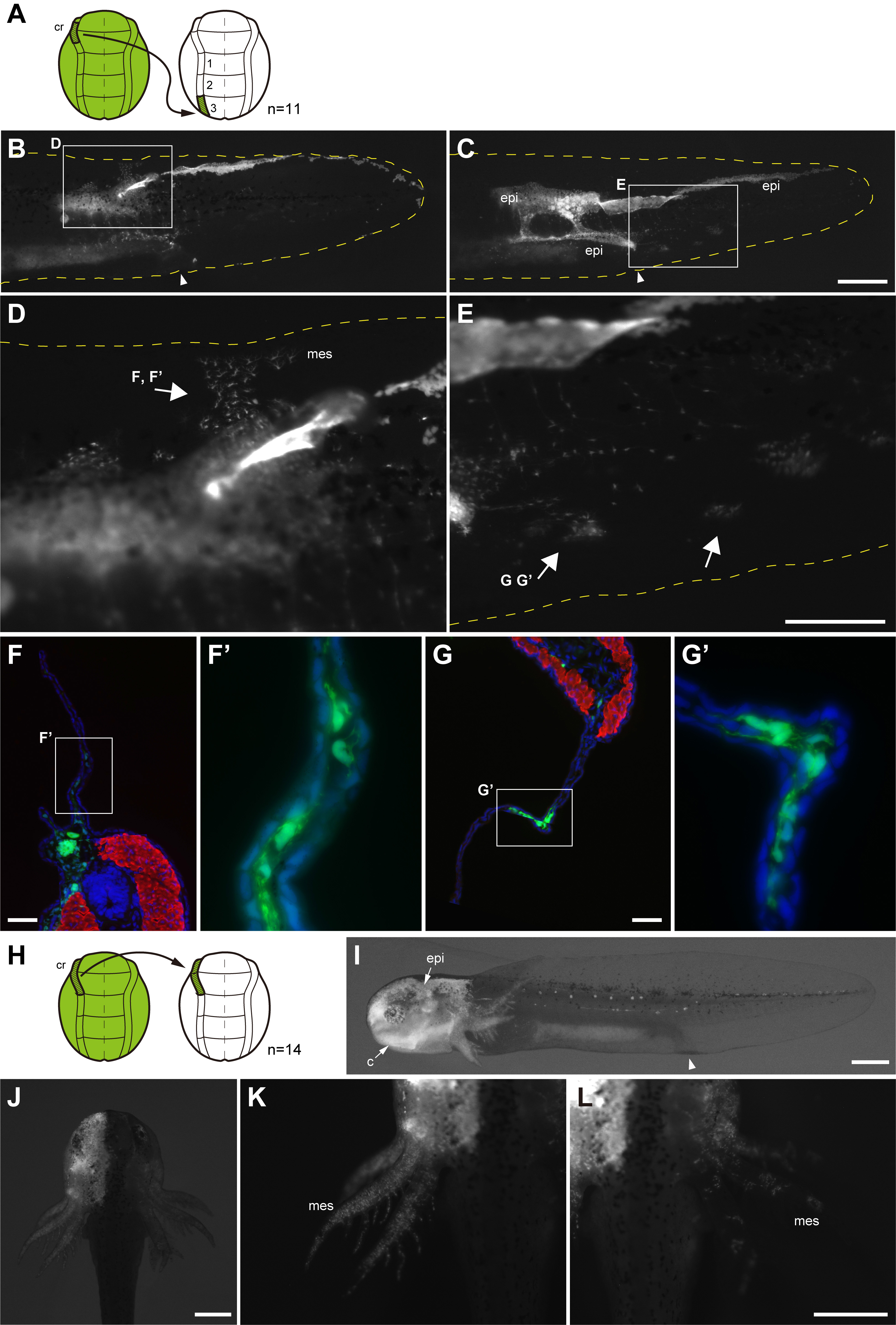
**

**Fig. S5: Grafting of cranial fold portions from GFP+ donors (stage 15) to white (d/d) hosts.** **A-G’,** a cranial neural fold fragment containing neural crest with prospective fate for the mandibular/hyoid arch was grafted heterotopically into the position of a host region3 trunk neural fold. Cranial neural crest cells give rise to mesenchymal cells both in the dorsal (B, D, F, F’) and ventral fin (C, E, G, G’) of the host. The mass of the GFP+ cranial tissue graft remains stationary forming also epidermis, pigment cells and, in the homotopic case, mesenchyme, cartilage, epidermis, pigment cells. Yellow dashed lines indicate shape of larva. White arrows indicate GFP+ fin mesenchyme. **F’ and G’**, higher enlargements of boxed areas in F and G. **H-L**, Homotopic implantation of an entire left GFP+ cranial fold fragment into a white host. **I**, Cranial neural crest cells give rise to components of lateral line nerves (glia and neuromasts) in trunk and tail and mesenchyme in the head and gills but do not migrate out posteriorly to form mesenchyme in the anterior trunk fin. **J**, dorsal view of head to anterior trunk area. **K and L**, enlargement of left and right gill region, respectively. White arrowhead points to position of cloaca. Number of experiments: A, 11; H, 14. Abbreviations: epi, epidermis; mes, mesenchyme; c, cartilage. Scale bars, 1 mm (C, I, J, K), 500 µ (E) and 100 µm (F, G).
